# Clinicopathological Significance of Cyclin-Dependent Kinase 2 (CDK2) in Ductal Carcinoma In Situ and Early-Stage Invasive Breast Cancers

**DOI:** 10.3390/ijms25095053

**Published:** 2024-05-06

**Authors:** Ayat Lashen, Shatha Alqahtani, Ahmed Shoqafi, Mashael Algethami, Jennie N. Jeyapalan, Nigel P. Mongan, Emad A. Rakha, Srinivasan Madhusudan

**Affiliations:** 1 Nottingham Biodiscovery Institute, School of Medicine, University of Nottingham, University Park, Nottingham NG7 3RD, UK; mzxal1@exmail.nottingham.ac.uk (A.L.); mzxsa20@exmail.nottingham.ac.uk (S.A.); msxas29@exmail.nottingham.ac.uk (A.S.); mszma4@exmail.nottingham.ac.uk (M.A.); plzjnj@exmail.nottingham.ac.uk (J.N.J.); svznpm@exmail.nottingham.ac.uk (N.P.M.); mrzear1@exmail.nottingham.ac.uk (E.A.R.); 2 Department of Pathology, Nottingham University Hospital, City Campus, Nottingham NG5 1PB, UK; 3 Faculty of Medicine and Health Sciences, University of Nottingham, Sutton Bonington Campus, Sutton Bonington LE12 5RD, UK; 4 Department of Pharmacology, Weill Cornell Medicine, New York, NY 10065, USA; 5 Department of Oncology, Nottingham University Hospitals, Nottingham NG5 1PB, UK

**Keywords:** CDK2, p53, CDK4, CDK6, breast cancer

## Abstract

Cyclin-dependent kinase 2 (CDK2) is a key cell cycle regulator, with essential roles during G1/S transition. The clinicopathological significance of CDK2 in ductal carcinomas in situ (DCIS) and early-stage invasive breast cancers (BCs) remains largely unknown. Here, we evaluated CDK2’s protein expression in 479 BC samples and 216 DCIS specimens. Analysis of CDK2 transcripts was completed in the METABRIC cohort (*n* = 1980) and TCGA cohort (*n* = 1090), respectively. A high nuclear CDK2 protein expression was significantly associated with aggressive phenotypes, including a high tumour grade, lymph vascular invasion, a poor Nottingham prognostic index (all *p*-values < 0.0001), and shorter survival (*p* = 0.006), especially in luminal BC (*p* = 0.009). In p53-mutant BC, high nuclear CDK2 remained linked with worse survival (*p* = 0.01). In DCIS, high nuclear/low cytoplasmic co-expression showed significant association with a high tumour grade (*p* = 0.043), triple-negative and HER2-enriched molecular subtypes (*p* = 0.01), Comedo necrosis (*p* = 0.024), negative ER status (*p* = 0.004), negative PR status (*p* < 0.0001), and a high proliferation index (*p* < 0.0001). Tumours with high *CDK2* transcripts were more likely to have higher expressions of genes involved in the cell cycle, homologous recombination, and p53 signaling. We provide compelling evidence that high CDK2 is a feature of aggressive breast cancers. The clinical evaluation of CDK2 inhibitors in early-stage BC patients will have a clinical impact.

## 1. Introduction

Cell cycle dysregulation is one of the hallmarks of cancer, resulting in uncontrolled cell proliferation. In normal breast epithelial cells, cell cycle progression is tightly controlled [1,2,3,4,5]. During the G1 phase of the cell cycle, the synthesis of essential proteins, transcripts, and organelles that are needed for DNA synthesis are completed. The cell then progresses to the S phase, where DNA replication occurs. During the G2 phase, microtubules are assembled, ready for cell division, which is completed during the M phase. When cells are deprived of mitogenic signals or fully differentiated, they enter a quiescent G0 phase. In the G0 phase of the cell cycle, E2F transcription factor is repressed by the retinoblastoma protein (Rb). Following mitogenic stimulation, the CDK3−cyclin C complex phosphorylates Rb at Ser807/811, thereby leading cells into G1. During early G1, D-type cyclins (e.g., cyclin D1) bind and activate CDK4 and/or CDK6. The CDK4/6-cyclinD1 complex then partially phosphorylates Rb, which results in the activation of E2F. E2F, whilst still bound to Rb, can transcribe several genes, including CCNE1 (cyclin E1), CCNA2, CCNB1, CDK2, and CDK1. During late G1, cyclin E binds to CDK2, which results in further phosphorylation of Rb, which releases and fully activates E2F. The transcription of S phase proteins by fully activated E2F prompts entry into the S phase of the cell cycle. The subsequent sustained phosphorylation of Rb by CDK2−cyclin A facilitates S/G2 transition, while CDK1−cyclin A allows for the commencement of mitosis, and by means of CDK1−cyclin B, it ensures progression through the M phase. The cells then return to G1 following the degradation of cyclin B and dephosphorylation of Rb by PP1 and PP2A phosphatases [1,2,3,4].

Besides playing a critical role in cell cycle progression, CDK2 is also involved in the DNA damage signalling response (DDR) in cells [6,7,8,9,10]. CDK2 has been shown to be involved during the homologous recombination (HR) pathway via activation of MRN-CtIP-BRCA1, which is essential for the repair of DNA double-strand breaks [11]. CDK2 can also regulate apoptosis via phosphorylation of FOXO1 [7] or MCL-1 [12]. In addition, CDK2 is also involved during signal transduction, DNA and RNA metabolism, and translation [13]. CDK2 can also interact with p53, a critical tumour-suppressing transcription factor [14,15]. The activation of p53 can repress the CDK2 transcript [14]. The ectopic CDK2 expression can also bypass p53-dependent senescence [16].

CDK2 may be involved in breast cancer pathogenesis. Preclinically, CDK2-deficient mice were shown to be resistant to cyclin E-mediated mammary tumours. Continuous treatment of a CDK2 inhibitor delayed the development of cyclin E-induced mammary tumour progression in that study [17]. More recently, hyperactive CDK2 has been described in triple-negative breast cancer cell lines [18]. However, the clinicopathological significance of CDK2 in clinical breast ductal carcinoma in situ (DCIS) or early invasive breast cancer (IBC) has not been described previously. Here, we have conducted a comprehensive study of the CDK2 protein expression in DCIS and IBC. Analyses of CDK2’s transcripts and bioinformatics were also completed in the METABRIC and TCGA cohorts, respectively.

## 2. Results

### 2.1. CDK2 Protein Expression in BC

**Figure 1:**
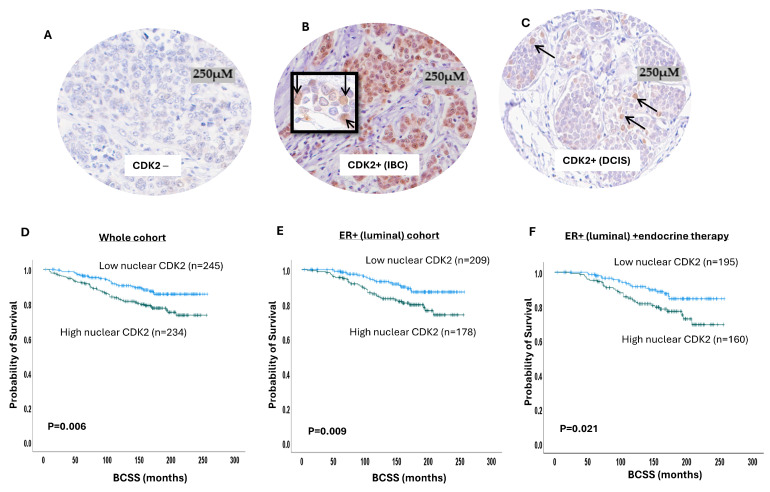
Cyclin-dependent kinase 2 (CDK2) expression in breast cancer (BC). Photomicrographs showing immunohistochemical staining of CDK2: (**A**) negative expression; (**B**) positive nuclear and cytoplasmic expression in invasive BC; (**C**) positive nuclear and cytoplasmic expression in ductal carcinoma in situ (DCIS); (**D**) Kaplan–Meier curve for CDK2 nuclear expression and breast cancer-specific survival (BCSS) in the whole cohort; (**E**) Kaplan–Meier curve for CDK2 nuclear expression and BCSS in luminal breast cancer; (**F**) Kaplan–Meier curve for CDK2 nuclear expression and BCSS in luminal breast cancer patients who received endocrine therapy.

Western blot showed specific bands for CDK2 protein at the predicted molecular weight (33 KDa). We observed nuclear and cytoplasmic expression of CDK2 (Figure 1A–C). A high nuclear expression of CDK2 was significantly associated with a larger tumour size (*p* = 0.03), higher tumour grade (*p* < 0.0001), low tubule formation (*p* = 0.006), increased nuclear pleomorphism (*p* < 0.0001), high mitosis (*p* < 0.0001), no-special-type (NST) tumour type (*p* < 0.0001), both HER2-enriched and triple-negative molecular subtypes (*p* < 0.0001), lymph node metastasis (*p* = 0.03), the presence of lympho-vascular invasion (*p* = 0.01), high-risk Nottingham Prognostic index (NPI) (*p* < 0.0001), and high Ki67 expression (*p* = 0.003) (Table 1). The cytoplasmic CDK2 expression did not show significant associations with clinicopathological parameters (Supplementary Table S3). We then proceeded to nuclear/cytoplasmic CDK2 co-expression analysis, and tumours with high nuclear and high cytoplasmic CDK2 co-expression were associated with a higher tumour grade (*p* < 0.0001), low tubule formation (*p* = 0.006), higher degree of nuclear pleomorphism (*p* < 0.0001) and high mitosis(*p* < 0.0001), NST tumour type (*p* < 0.0001), HER2-enriched molecular subtype (*p* < 0.0001), presence of lympho-vascular invasion (*p* = 0.01), moderate and poor NPI groups (*p* < 0.0001), and higher Ki67 expression (*p* = 0.021) (Supplementary Table S4).

In terms of patient outcome, there was a significant association between high nuclear CDK2 and poor breast cancer-specific survival (BCSS) (*p* = 0.006) (Figure 1D), while cytoplasmic CDK2 did not show association with the patient outcome (Supplementary Figure S1B). In luminal BC, high nuclear CDK2 was significantly associated with poor BCSS (*p* = 0.009) (Figure 1E). In patients who received endocrine therapy, high nuclear CDK2 was linked to a poor patient outcome (*p* = 0.02) (Figure 1F) but not in endocrine-therapy-naïve patients (*p* = 0.59) (Supplementary Figure S1C). In triple-negative (Supplementary Figure S1D) or HER2-enriched (Supplementary Figure S1E) subtypes, the CDK2 expression did not influence survival. A high nuclear with low cytoplasmic co-expression also showed an association with a worse outcome (*p* = 0.02) (Supplementary Figure S1F). In the multivariate analysis, CDK2 was independently associated with a poor survival outcome (*p* = 0.02) in relation to tumour size and stage (Table 2). Taken together, the data provide the first comprehensive evidence that CDK2 is a prognostic and predictive biomarker in early-stage sporadic BC.

### 2.2. Nuclear CDK2/p53 Co-Expression

**Figure 2:**
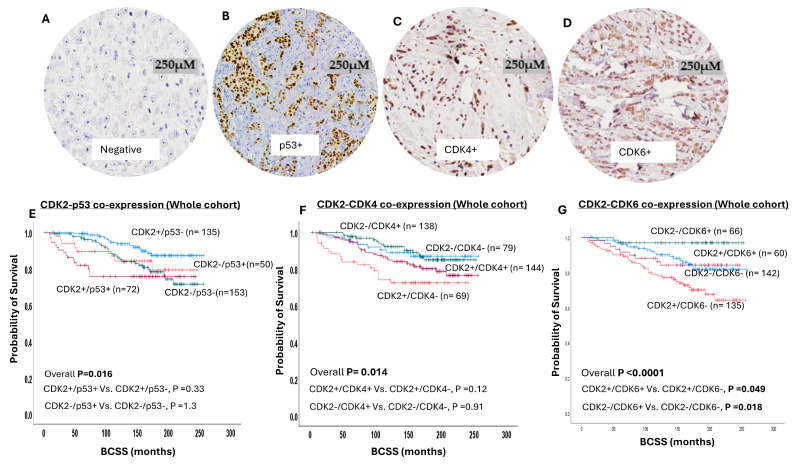
Cyclin-dependent kinase 2 (CDK2) co-expression with P53, cyclin-dependent kinase 4 (CDK4), and cyclin-dependent kinase 6 (CDK6) in breast cancer (BC). (**A**) Negative immunohistochemical expression of CDK4. (**B**) Positive immunohistochemical nuclear expression of p53 in invasive BC. (**C**) Positive immunohistochemical nuclear expression of CDK4 in invasive BC. (**D**) Positive immunohistochemical nuclear expression of CDK6 in invasive BC. (**E**) Kaplan–Meier curve for CDK2/p53 co-expression and breast cancer-specific survival (BCSS) in whole cohort, which shows significant association between CDK2+/p53+ and shortest survival. (**F**) Kaplan–Meier curve for CDK2/CDK4 co-expression and BCSS in whole cohort, which shows significant association between CDK2+/CDK4- and shortest survival. (**G**) Kaplan–Meier curve for CDK2/CDK6 co-expression and BCSS in whole cohort, which shows significant association between CDK2+/CDK6- and shortest survival.

Somatic p53 mutation and activation is common in BC [19]. p53 is essential for cell cycle regulation. Moreover, there is evidence of an interaction between CDK2 and p53 [14,15]. We therefore conducted nuclear CDK2-p53 co-expression analysis (Figure 2A,B). A high nuclear CDK2/high p53 co-expression showed a significant association with a younger patient age (*p* = 0.03), high tumour grade (*p* < 0.0001), low tubule formation (*p* = 0.006), high degree of nuclear pleomorphism (*p* < 0.0001) and high mitosis (*p* < 0.0001), NST tumour type (*p* < 0.0001), HER2-enriched molecular subtype (*p* < 0.0001), lymph node metastasis ( *p* = 0.04), presence of lympho-vascular invasion (*p* = 0.03), poor NPI groups (*p* < 0.0001), and high Ki67 expression (*p* < 0.0001) (Supplementary Table S5).

In terms of the patient outcome, CDK2/p53 co-expression influences the survival outcome (overall *p* = 0.01) (Figure 2E), and this association was maintained in luminal BC (Supplementary Figure S2A) and in endocrine-therapy-treated patients (Supplementary Figure S2B). However, no association was observed between CDK2 with p53 co-expression and the outcome in either endocrine-therapy-naïve (Supplementary Figure S2C) or triple-negative (Supplementary Figure S2D) or HER2-enriched BC (Supplementary Figure S2E) patients.

The sequential activation of CDK4/6 and CDK2 is essential for the progression of cells from the G1 to the S phase of the cell cycle [1,2,3,4]. We therefore immunohistochemically stained CDK4 (Figure 2C) and CDK6 (Figure 2D) and conducted co-expression analysis with CDK2. (Figure 2E–G).

### 2.3. CDK2-CDK4 Co-Expression

Western blot showed specific bands for CDK4 protein at the predicted molecular weight (34 KDa). A high nuclear CDK2/low nuclear CDK4 co-expression showed a significant association with a younger patient age (*p* = 0.03), high tumour grade (*p* < 0.0001), low tubule formation (*p* = 0.004), high degree of nuclear pleomorphism (*p* < 0.0001) and high mitosis (*p* < 0.0001), NST tumour type (*p* < 0.0001), HER2-enriched molecular subtype (*p* < 0.0001), poor NPI groups (*p* < 0.0001), and high Ki67 expression (*p* < 0.0001) (Supplementary Table S6). In terms of the patient outcome, CDK2/CDK4 co-expression influences the survival outcome (overall *p* = 0.01) (Figure 2F), and this association was maintained in luminal BC (*p* = 0.033) (Supplementary Figure S3B). However, no association was observed between CDK2 with nuclear CDK4 co-expression and the outcome in endocrine-therapy-treated patients (Supplementary Figure S3C) or endocrine-therapy-naïve patients (Supplementary Figure S3D), or in triple-negative (Supplementary Figure S3E) or HER2-enriched BC (Supplementary Figure S3F).

### 2.4. CDK2-CDK6 Co-Expression

Western blot showed specific bands for CDK4 protein at the predicted molecular weight (34 KDa). A high nuclear CDK2/low nuclear CDK6 co-expression showed a significant association with a younger patient age (*p* = 0.035), high tumour grade (*p* < 0.0001), low tubule formation (*p* = 0.015), high degree of nuclear pleomorphism (*p* < 0.0001) and high mitosis (*p* < 0.0001), NST tumour type (*p* < 0.0001), presence of lympho-vascular invasion (*p* = 0.01), luminal B subtype (*p* < 0.0001), poor NPI groups (*p* < 0.0001), and high Ki67 expression (*p* < 0.0001) (Supplementary Table S7). In terms of the patient outcome, CDK2/CDK6 co-expression influences the survival outcome (*p* < 0.0001) (Figure 2G), and this association was maintained in luminal BC (*p* < 0.0001) (Supplementary Figure S4B), including endocrine-therapy-treated patients (Supplementary Figure S4C), but not in endocrine-therapy-naïve patients (Supplementary Figure S4D). However, no association was observed between CDK2 with nuclear CDK6 co-expression and outcome in either triple-negative (Supplementary Figure S4D) or HER2-enriched BC (Supplementary Figure S4D).

### 2.5. CDK2 in Pre-Invasive DCIS

Preclinical studies revealed that CDK2-deficient mice are resistant to cyclin E-mediated mammary tumours, suggesting a role for CDK2 in early BC pathogenesis. Here, we investigated the CDK2 protein expression in pre-invasive DCIS [17]. In the DCIS cohort, we also observed both nuclear and cytoplasmic expressions of CDK2. A high nuclear CDK2 expression showed a significant association with a negative PR status (*p* = 0.009) and high Ki67 expression (*p* < 0.0001) (Supplementary Table S8). On the other hand, a low cytoplasmic CDK2 expression was associated with a high tumour grade (*p* < 0.0001), triple-negative and HER2-enriched molecular subtypes (*p* = 0.01), Comedo necrosis (*p* = 0.002), negative ER status (*p* = 0.01), and negative PR status (*p* = 0.002) (Supplementary Table S9). In addition, a high nuclear/low cytoplasmic co-expression showed a significant association with a high tumour grade (*p* = 0.043), triple-negative and HER2-enriched molecular subtypes (*p* = 0.01), Comedo necrosis (*p* = 0.024), negative ER status (*p* = 0.004), negative PR status (*p* < 0.0001), and high Ki67 expression (*p* < 0.0001) (Supplementary Table S10).

In terms of the patient outcome, no association was observed between either nuclear or cytoplasmic CDK2 or their co-expression and local recurrence-free survival (Supplementary Figure S5A–C).

### 2.6. CDK2 Transcripts in BC

**Figure 3:**
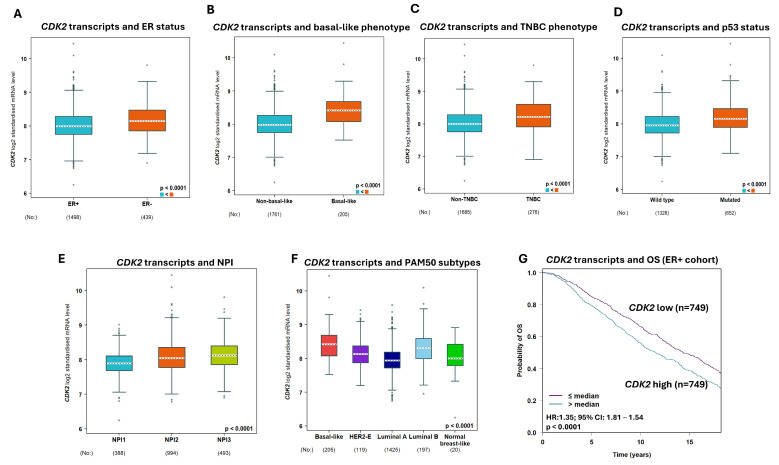
The association of *Cyclin-dependent kinase 2* (*CDK2*) transcript with breast cancer (BC). (**A**) *CDK2 mRNA* expression showed a significant association with negative oestrogen receptor (ER) status. (**B**) *CDK2 mRNA* expression has a significant association with the basal-like BC phenotype. (**C**) The association of *CDK2 mRNA* expression with the triple-negative BC phenotype. (**D**) *CDK2 mRNA* expression had a strong association with p53 status (wild-type vs. mutated). (**E**) CDK2 mRNA expression showed a significant association with the Nottingham prognostic index (NPI). (**F**) The association of *CDK2 mRNA* expression with PAM50 molecular phenotypes. (**G**) The Kaplan–Meier curve showed a strong association between a high *CDK2 mRNA* expression and poor overall survival in the ER+ cohort.

A high *CDK2 mRNA* level was highly associated with ER- BC (*p* < 0.0001), basal-like BC (*p* < 0.0001), triple-negative phenotype (*p* < 0.0001), p53 mutant (*p* < 0.0001), high-risk NPI phenotypes (*p* < 0.0001) (Figure 3A–F), and poor survival (Figure 3G) in the ER+ cohort.

### 2.7. Bioinformatics

**Figure 4:**
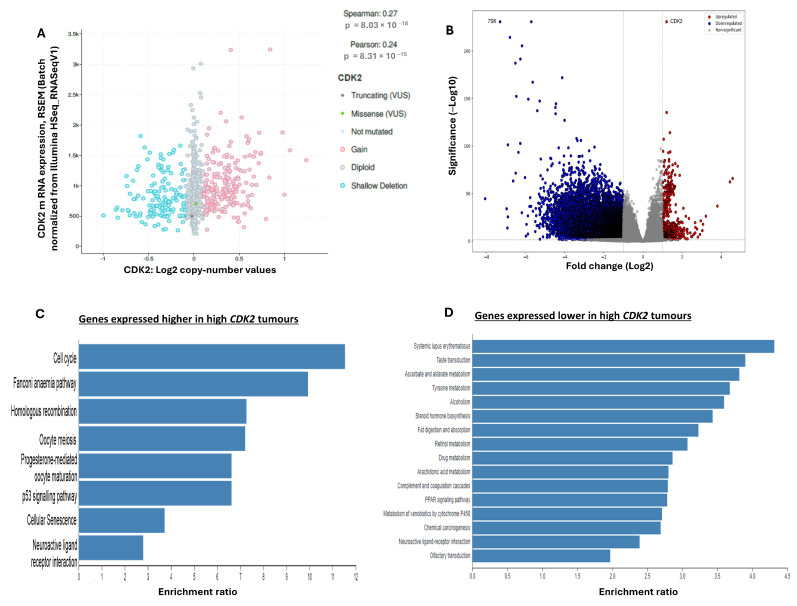
Pathways associated with high *Cyclin-dependent kinase 2* (*CDK2*) mRNA expression in breast cancer tumours. (**A**) CBioportal GISTIC analysis of CDK2 shows weak positive correlation between copy number alterations and mRNA levels. (**B**) Volcano plot of differentially expressed genes between Q1 (tumours low in *CDK2*) and Q4 (tumours with high *CDK2*). Genes expressed higher in Q4 are shown in red, and genes expressed lower in Q4 are shown in blue. Pathways associated with differentially expressed genes were (**C**) higher and (**D**) lower in tumours with high *CDK2* (Q4).

We investigated the significance of CDK2 in breast cancer utilising CBioportal. The TCGA BRCA cohort of 994 samples showed only two mutations in *CDK2* (V123Sfs*21 frameshift deletion and V230F missense). Interestingly, copy number alterations consisted of gains and shallow deletions, while variations in expression within the diploid samples were detected, therefore showing a low but significant positive correlation (Pearson correlation coefficient 0.24, *p* < 0.001; 960 samples; Figure 4A). Differential analysis was then performed on the TCGA BRCA cohort of 1080 samples. *CDK2*-low-expressing tumours (272 samples, Quartile 1) were compared to *CDK2*-high-expressing tumours (272 samples, Quartile 4). There were 494 genes that were expressed higher in high-*CDK2* tumours, and 10,245 genes were expressed lower in high-*CDK2* tumours with a log2 fold change ≥ 1 and FDR *p*-value < 0.05 (Figure 4B; Supplementary File S1). *CDK2* exhibited a significant difference of log2 FC 1.2 between Q1 and Q4. Genes that were expressed higher or lower in tumours with high CDK2 were identified by pathway analysis. Significant pathways for genes that were expressed higher with high CDK2 were involved in the cell cycle, homologous recombination, and p53 signalling pathway (Figure 4C). The genes in the cell cycle included cyclins CCNE1 and CCNA1, CDK1, PLK1, cdc25A, BUB1, MCM2, MCM4, and MCM8, showing the cell cycle progressing from the G1, G2, and M phases. This could therefore be linked with greater proliferation in these tumours. With greater proliferation comes genome instability, and CDK2 is also known to have a role in DNA damage to the check point. We could observe that BRCA1 and BLM were also expressed higher in high-CDK2 tumours. Interestingly, the gene that showed the lowest expression in high-CDK2 tumours was the non-coding 7SK RNA/RF00100 [20], which has been implicated in the inhibition of cell proliferation (Figure 4B). The pathways that were seen for the lower expressed genes for Q4 were UDP-Glucuronosyltransferases, which are involved in many pathways, including drug metabolism, drug resistance, and cancer progression [21] (Figure 4D). The correlation between *CDK2* and *CDK4/6* or *TP53* showed a significant positive correlation (*CDK4,* r = 0.43, *p* < 0.001; *CDK6*, r = 0.15, *p* < 0.001 and *TP53*, r = 0.39, *p* < 0.001).

## 3. Discussion

Cyclin-dependent kinase 2 (CDK2) is a critical cell cycle regulator of G1/S [1,2,3,4,5]. CDK2 is also involved during DDR, HR, signal transduction, apoptosis, and DNA and RNA metabolism in cells [6,7,8,9,10,13]. Here, we comprehensively evaluated CDK2 in BC. In invasive BC, high nuclear CDK2 was associated with an aggressive pathology, as well as poor survival outcomes. In the DCIS cohort, high nuclear CDK2 protein was associated with aggressive pathology features but not with survival, which was likely related to the lower number of patients in this cohort. Larger studies will be required to evaluate the prognostic significance of CDK2 in DCIS. Moreover, a limitation to the current study is that it is retrospective. Prospective clinical studies will be required to confirm our findings. A high *CDK2* transcript was also associated with aggressive phenotypes and poor survival outcomes. Tumours with high *CDK2* transcripts also have a higher expression of genes involved in the cell cycle, HR, and p53 signalling. Taken together, our data provide clinical evidence that CDK2 is involved in BC’s pathogenesis and prognosis. Our data also suggest that CDK2 targeting by small molecule inhibitors might have a clinical impact in early-stage BC.

The prognostic and/or predictive significance of CDK2 has been described in other solid tumours [13]. In Glioblastomas, CDK2 was required for proliferation and associated with poor patient survival [22]. CDK2 induced radio resistance, and its depletion promoted radiosensitivity in that study. Moreover, a CDK2 inhibitor also attenuated tumour glioblastoma growth in vitro and in vivo [22]. CDK2 overexpression has also been shown in colorectal cancers compared to benign adenomas [23]. In acute myeloid leukaemia, the up-regulation of HDAC3-AKT-P21-CDK2 signalling has been associated with poor survival [24]. In bladder cancer, an increased level of CDK2 was associated with an advanced tumour stage and grade [25]. The up-regulation of MTHFD2, which binds to CDK2, was previously shown to be associated with a high grade, advanced stage, and poor survival in BC [26].

The tumour-suppressing transcription factor p53 is a critical regulator of the G1/S and G2/M phases of the cell cycle [19]. Mutations in p53 are seen in 17% of luminal-A BCs, 41% of luminal -B BCs, 50% of HER-2-overexpressing BCs, and 88% of basal-like BCs. An advanced stage, high grade, and poor survival are features of p53-mutant BC [27]. Interestingly, CDK2 can also interact with p53 [14,15]. The activation of p53 can repress the CDK2 transcript [14]. An ectopic CDK2 expression can also bypass p53-dependent senescence [16].

Although CDK2 controls the G1/S transition and promotes DNA replication, it is dispensable for cell cycle progression due to redundancy with CDK1 [28]. However, CDK2 also has non-redundant functions that can be revealed in certain genetic backgrounds, and it was reported to promote the G2/M DNA damage response checkpoint in TP53 (p53)-deficient cancer cells [28]. In the current study, we have shown that p53-mutant BCs with high CDK2 expressions are particularly aggressive and linked with poor survival, including in luminal BCs that received endocrine therapy. Interestingly, in p53 wild-type BCs with low CDK2, we found that this group showed the shortest survival after 15 years. Previous studies show that death due to BC usually occurs in the first 10 years, and after this period, the risk of death due to BC is lower [29]. When the analysis was restricted to p53-mutant BCs with high CDK2 expressions and p53 wild-type BCs with low CDK2, we found significant differences in patient outcomes between both groups.

The clinical use of CDK4/6 inhibitors has significantly improved survival outcomes in ER+ advanced or early-stage BC [30,31,32,33,34,35,36]. However, the prognostic and/or predictive significance of CDK2 expression in CDK4- or CDK6-expressing BCs has not been described previously. Here, we provide the first clinical evidence that CDK2 overexpression in CDK4/6-negative tumours is significantly linked with aggressive tumours and poor survival compared to tumours with low CDK2 and highly CDK4/6-expressing tumours. The data suggest that CDK2 targeting in low-CDK4/6 tumours may be a clinical strategy.

The clinical development of CDK2 small molecule inhibitors is an area of intense research [13]. CDK2/cyclin E1 (CCNE1)-expressing cell lines are sensitive to INCB123667, a highly potent CDK2 inhibitor that has recently entered phase 1 clinical trials [37]. As Cyclin E amplification and overexpression may drive CDK4/6 resistance in patients, it is anticipated that INCB123667 will also have activity in patients developing intrinsic or acquired resistance to CDK4/6 inhibitors [37]. In addition, there is also an opportunity for synthetic lethality, particularly in CCNE1-amplified tumours that are addicted to CDK2-dependent pathways [37]. Other highly potent CDK-2-selective inhibitors that are undergoing phase I trial investigation in cancers include PF-07104091 [38] and BLU-222 [39].

## 4. Conclusions

Our data taken in their entirety provide clear evidence that CDK2 targeting may have a clinical impact in p53-mutant and low-CDK4/6-expressing BC. Pre-clinical and clinical evaluation of these approaches will be required to develop CDK2 targeting as a novel new precision oncology approach in BCs.

## 5. Materials and Methods

### 5.1. BC Cell Lines

MCF7 and T47D were purchased from the American Type Culture Collection (ATCC, Manassas, VA, USA). MDA-MB-231, MCF7, and T47D were grown in RPMI (R8758, Merck, Feltham, UK) supplemented with 10% foetal bovine serum (F4135, Merck, UK) and 1% Penicillin–Streptomycin (P4333, Merck, UK). MCF10A is a normal breast epithelial cell line. The MCF10DCIS BC cell line was previously derived from a xenograft originating from premalignant MCF10AT cells injected into SCID mice [40,41]. Injection of the MCF10DCIS cells into SCID mice results in a predominantly comedo DCIS phenotype [40,41]. MCF10A and MCF10DCIS cells (DCIS cell line) were cultured in DMEM-F12 supplemented with 10% horse serum, 5 mg/mL insulin, 1 mg/mL cholera toxin, 100 mg/mL EGFR, 5 mg/mL hydrocortisone, and 1% penicillin–streptomycin. Cell lines were routinely tested for mycoplasma contamination every 4 weeks.

### 5.2. Western Blots

Cells were harvested and lysed in RIPA buffer (R0278, Sigma, Coventry, UK) with the addition of a protease cocktail inhibitor (P8348, Sigma, UK), phosphatase inhibitor cocktail 2 (P5726, Sigma, UK), and phosphatase inhibitor cocktail 3 (P0044, Sigma) and stored at −20 °C. Proteins were quantified using a BCA Protein Assay kit (23225, Thermofisher, Loughborough, UK). Samples were run on SDS-bolt gel (4–12%) bis-tris. Membranes were incubated with primary antibodies as follows: CDK2 (1:1000, ab32147 Abcam), CDK4 (1:200, DCS-31, Invitrogen), and CDK6 (1:1000, SD20-50, Invitrogen). Membranes were then washed and incubated with Infrared dye-labelled secondary antibodies (LiCor) [IRDye 800CW Donkey Anti-Rabbit IgG (926-32213) and IRDye 680CW Donkey Anti-Mouse IgG (926-68072)] at a dilution of 1:10,000 for 1 h. Membranes were scanned with a LiCor Odyssey machine (700 and 800 nm) to determine protein levels. Anti-β-actin primary antibody (Sigma-Aldrich) was used as a loading control (1: 15,000). The specific bands for CDK2, CDK4, and CDK6 were detected (Supplementary Figures S1A, S3A and S4A).

### 5.3. Clinically Invasive BC Cohorts

CDK2’s protein expression was evaluated on a well-characterised primary BC series (*n* = 479) from patients treated at Nottingham City Hospital, NHS Trust, Nottingham, United Kingdom. Clinical and tumour characteristics, including patient’s age at diagnosis, histological tumour type, grade, and grade components including nuclear pleomorphism, mitosis and tubule formation, tumour size, lymph node status, Nottingham Prognostic Index (NPI), and lympho-vascular invasion (LVI), were available (Supplementary Table S1) as described previously [42]. In addition, the outcome data in the form of BC-specific survival (BCSS), defined as the time (in months) from the date of primary surgical treatment to the time of death by BC, was also collected from patients` records. Adjuvant treatment was given according to the institutional protocols. Information regarding oestrogen receptor (ER), progesterone receptor (PR), and human epidermal growth factor 2 (HER2) expression is available from previous publications from our group [43,44,45,46]. Tumours were also classified based on ER, PR, and HER2 into three molecular subtypes (luminal, triple-negative (TN), and HER2-enriched) as follows: ER+ HER2- (luminal BC), ER-, PR-, and HER2- (TNBC) tumours, as well as tumours with HER2+ (HER2-enriched) [47].

### 5.4. Pre-Invasive BC Cohort

CDK2 protein expression was also evaluated on a well-characterised cohort (*n* = 217) of ductal carcinoma in situ from patients treated at Nottingham City Hospital, NHS Trust, Nottingham, United Kingdom (Supplementary Table S2).

In view of the critical role of p53 in the progression of BC [27,48,49,50] and its interaction with CDK2 [14,15], the data regarding immunohistochemical expression were used for comparative analysis with CDK2 and were available from a previous study. An H-score of 10 was used to categorise P53 nuclear expression as negative (wild-type P53) and positive (mutated p53) [51]. For the importance of Ki67 as a surrogate of cellular proliferation in BC and its role in the cell cycle, Ki67 levels as measured in a previous study were used in comparative analysis with CDK2 [52,53].

### 5.5. CDK2, CDK4, CK6, and p53 Protein Expression Evaluation

Tumour samples were arrayed using the Grand Master^®^ (3D HISTECH^®^, Budapest, Hungary) as described previously [51]. Tissue sections of 4 μm thicknesses were dewaxed using the Novocastra Novolink™ Polymer Detection Systems kit (Code: RE7280-K, Leica, Biosystems, Newcastle, UK), and endogenous peroxidase activity was blocked with 0.3% hydrogen peroxide in methanol for 10 min. Antigen retrieval was performed in citrate buffer, pH 6.0, using a microwave (Whirlpool JT359 Jet Chef 1000 W) for 20 min. Mouse monoclonal CDK2 were placed in Leica antibody diluent (RE AR9352, Leica, Biosystems, UK) and incubated for 60 min at room temperature. The sections were counterstained with haematoxylin. CDK4 and CDK6 were diluted at 1:20 and 1:15, respectively, and were incubated for 1 h at room temperature. Diaminobenzidine (DAB) was used to visualise the immunochemical staining, and finally, counterstaining with Meyer’s Haematoxylin was performed.

The CDK2 protein showed both nuclear and cytoplasmic expression. Therefore, the percentage of positive tumour cells was evaluated for both compartments. A semi-quantitative modified histochemical score (H-score) method was used to assess both markers, and the final H-score was obtained by giving a range of 0 to 300 as previously described [54,55]. X-tile bioinformatics software version 3.6.1 (School of Medicine, Yale University, New Haven, CT, USA) was used [56] to categorise CDK2’s H-scores into low and high expression. This software randomly divides the patient cohort into two separate equal sets, training and validation sets, by producing separate lists of ‘censored’ and ‘uncensored’ observations, ranked by patients’ follow-up time [56]. The optimal cutoffs were determined by locating the brightest pixel on the X-tile plot diagram of the training set [56]. Statistical significance was tested by applying the obtained cutoff to the validation set. H-scores of 5 and 70 were considered the best cut-offs of nuclear and cytoplasmic expressions of CDK2, respectively. The CDK2 expressions were compared with the available clinicopathological parameters and with the outcome data. CDK4 and CDK6 antibodies showed both nuclear and cytoplasmic expression. An H-score was calculated for the nuclear expression. Using X tile, H-scores of 110 and 80 were considered the best cut-offs of nuclear CDK4 and nuclear CDK6, respectively.

Immunostaining for p53 showed nuclear expression, and its score was evaluated using the H-score. A 10% cut-off was used as the optimal cut-off of categorisation of p53 expression into negative (wild-type) and positive (mutant) tumours based on X tile. For the interactions between CDK2, p53, CDK4, and CDK6 markers, the whole cohort was categorised into 4 groups. These groups were assessed against the clinicopathological parameters and patient outcomes.

This study was approved by the Yorkshire & the Humber—Leeds East Research Ethics Committee (REC Reference: 19/YH/0293) under the IRAS Project ID: 266925. Data collected were fully anonymised. Written informed consent was obtained from the patients

### 5.6. Bioinformatics

CBioportal was used for evaluating the copy number variation against mRNA levels (RNA-seq data) in the breast cancer TCGA cohort (TCGA, Firehose Legacy, 960 samples/patients) [57]. Utilising RNA-Seq data from GDC (https://portal.gdc.cancer.gov/, accessed on 31 March 2023), the TCGA_BRCA cohort (1080 samples) was split into quartiles depending on the CDK2 expression values. Quartile 1 (Q1) contained low-CDK2 tumours, and quartile 4 (Q4) contained high-CDK2 tumours. Differential analysis between CDK2 Q1 and Q4 was performed using DESeq2 [58]. Differentially expressed genes were significant if they met a threshold of log2 FC ≥ 1 and FDR-corrected *p*-value < 0.05. Pathway analysis was performed using WebGestalt v2019 [59]. Over-representation analysis was performed on Q1 highly expressed genes and Q4 highly expressed genes separately. Significant pathways were FDR-corrected <0.05.

The prognostic significance of CDK2’s mRNA levels was also evaluated in the METABRIC (Molecular Taxonomy of Breast Cancer International Consortium) cohort of 1980 BCs [60]. The gene expression dataset was publicly available at bc-GenExMiner (http://bcgenex.ico.unicancer.fr/BC-GEM/GEM-Accueil.php?js=1) for the analysis.

### 5.7. Statistical Analysis

Statistical Package for the Social Sciences software v.27.0 (SPSS, Chicago, IL, USA) was used for statistical analysis. Chi-square test was used for analysis of categorical data. Outcome analysis was assessed using Kaplan–Meier curves and the log-rank test. The association of CDK2 with the different molecular classes of BC was also evaluated. Cox regression models were used for the multivariate analysis. For statistical analysis, the Ki67 expression levels were categorised into low and high proliferative tumours based on a 14% cut-off [52,53]. Estimated hazard ratios (HRs) and their 95% confidence intervals (95% CIs) were calculated. For all tests, *p* < 0.05 (two-tailed) was statistically significant.

## Figures and Tables

**Table 1:** Relationship between nuclear CDK2 and clinicopathological parameters in BC.

Variables	Nuclear CDK2 Expression		X^2^ *p*-Value
	Negative/Low H. Score ≤ 5	Positive/High H. Score > 5	
Age at diagnosis (years)			
<50	63 (46%)	74 (54%)	2.1
≥50	182 (53%)	160 (47%)	0.1
Menopausal state			
Premenopausal	78 (50%)	78 (50%)	0.1
Post-menopausal	167 (52%)	156 (48%)	0.7
Tumour size (cm)			
≤2	159 (55%)	130 (45%)	4.3
>2	86 (45%)	104 (55%)	**0.03**
Histologic tumour grade			
Grade 1	58 (69%)	26 (31%)	
Grade 2	120 (60%)	80 (40%)	39.1
Grade 3	67 (34%)	128 (66%)	**<0.0001**
Tubule formation			
1	29 (74%)	10 (26%)	
2	71 (53%)	64 (47%)	10.1
3	145 (47%)	160 (53%)	**0.006**
Nuclear Pleomorphism			
1	3 (43%)	4 (57%)	
2	103 (65%)	55 (35%)	18.6
3	139 (44%)	175 (56%)	**<0.0001**
Mitosis			
1	165 (64%)	94 (36%)	
2	39 (44%)	50 (56%)	38.9
3	41 (31%)	90 (69%)	**<0.0001**
Histologic tumour types			
No special type (NST)	133 (44%)	167 (56%)	
Lobular	29 (57%)	22 (43%)	
Other special types	15 (62%)	9 (38%)	15.916
NST mixed	68 (65%)	36 (35%)	**0.001**
Molecular subtypes			
Luminal A	115 (63%)	68 (37%)	
Luminal B	64 (45%)	77 (55%)	
HER2-enriched.	8 (38%)	13 (62%)	18.7
Triple-negative	23 (36%)	40 (64%)	**<0.0001**
Lymph node invasion			
Absent	171 (55%)	138 (45%)	6.1
Present	74 (44%)	96 (56%)	**0.013**
Lympho-vascular invasion			
Absent	193 (55%)	160 (45%)	6.7
Present	52 (41%)	74 (59%)	**0.01**
Nottingham prognostic index			
Good prognostic group	113 (66%)	59 (34%)	
Moderate prognostic group	109 (46%)	130 (54%)	25.7
Poor prognostic group	23 (34%)	45 (66%)	**<0.0001**
Ki67 index			
Low ≤10%	121 (60%)	80 (40%)	9.1
High >10%	67 (44%)	85 (56%)	**0.003**

HER2, human epidermal growth factor receptor 2. Significant *p*-values are in bold. Ki67 expression levels were classified into low and high based on 10% cutoff.

**Table 2:** Multivariate analysis.

Parameters	BCSS		
	Hazard Ratio	95% (CI)	*p*-Value
CDK2	1.7	1.1-2.7	0.028
Tumour size	1.7	1.1-2.8	0.027
Stage	1.8	1.3-2.4	<0.0001

BCSS, breast cancer-specific survival. 95%CI, 95% confidence interval. Significant *p*-values are in bold.

## Data Availability

Data supporting the study can be found in the Supplementary Materials File, and the corresponding author can make any materials available upon request. Aggregate data from the referenced datasets are available from the corresponding author on reasonable request.

## Supplementary Materials

The following supporting information can be downloaded at https://www.mdpi.com/article/10.3390/ijms25095053/s1.
