# Supplementary material for: Clinicopathological Significance of Cyclin-Dependent Kinase 2 (CDK2) in Ductal Carcinoma In Situ and Early-Stage Invasive Breast Cancers
Source: Int J Mol Sci. 2024 May 6;25(9):5053. doi: 10.3390/ijms25095053 (PMC11084890; doi:10.3390/ijms25095053)
Supplement: Supplementary file 1 [file ijms-25-05053-s001.zip › Supplementary materials_revision2.pdf]

Supplementary Table S1. Clinicopathological characteristics of the invasive breast cancer cohort

Variables	No (%)
**Age at diagnosis**	
<50	137 (29%)
≥50	342 (71%)
**Menopausal status**	
Premenopausal	156 (32%)
Post-menopausal	323 (68%)
**Tumour size Groups**	
≤ 2cm	289 (60%)
> 2cm	190(40%)
**Grade**	
Grade 1	84 (17%)
Grade 2	200 (42%)
**Grade 3**	195 (41%)
**Tubule**	
1	39 (8%)
2	135 (28%)
3	305 (64%)
**Polymorphism**	
1	7 (1%)
2	158 (33%)
3	314 (66%)
**Mitosis**	
1	259 (54%)
2	89 (19%)
3	131 (27%)
**Histologic tumour types**	
No Special Type (NST)	300 (62%)
Lobular	51 (11%)
Other special types	24 (5%)
NST mixed	104 (22%)
**Lymhovascular invasion**	
Absent	353 (74%)
Present	126 (26%)
**Lymph node status**	
Absent	309 (65%)
Present	170 (35%)
**Nottingham prognostic index**	
Good prognostic group	172 (36%)
Moderate prognostic group	239 (50%)
Poor prognostic group	68 (14%)
**Ki67 expression**	
Low ≤ 14%	201 (57%)
High>14%	152 (43%)
**Molecular subtypes**	
Luminal A	183 (45%)
Luminal B	141 (35%)
HER2 enriched.	21 (5%)

Triple negative	63 (15%)

Supplementary Table S2. Clinicopathological characteristics of the DCIS cohort

Categories	Number (percentage)
**Age**	
<50	59 (28%)
≥50	158 (72%)
**Tumour size**	
≤2cm	88 (40%)
>2cm	129 (60%)
**Tumour grade**	
Low grade	29 (13%)
Intermediate grade	63 (29%)
High grade	125 (58%)
**Molecular subtype**	
Luminal A	90 (55%)
Luminal B	30 (19%)
HER2+	27 (17%)
Triple Negative	15 (9%)
**Comedo necrosis**	
Negative	73 (34%)
Positive	144 (66%)
ER Status	
Negative	45 (24%)
Positive	139 (76%)
**PR Status**	
Negative	72 (39%)
Positive	114 (61%)
**HER2 Status**	
Negative	86 (80%)
Positive	21 (20%)
**Recurrence**	
No recurrence	194 (89%)
Recurrence	23 (11%)
**Ki67 expression**	
Low (≤14)	134 (75%)

High (>14)	45 (25%)

HER2, human epidermal growth factor receptor 2 Significant P values are in bold.

Supplementary Table S3. Relationship between cytoplasmic CDK2 and clinicopathological parameters in invasive breast cancer.

**Variables**	**Cytoplasmic CDK2 expression**		**X²**
	Negative /low H. score ≤70	Positive/ high H. score >70	***P*-value**
**Age at diagnosis (years)**			
<50	70 (51%)	67 (49%)	0.06
≥50	179 (52%)	163 (48%)	0.8
**Menopausal state**			
Premenopausal	80 (51%)	76 (49%)	0.04
Post-menopausal	169 (52%)	154 (48%)	0.83
**Tumour size (cm)**			
≤2	150 (52%)	139 (48%)	0.002
>2	99 (52%)	91 (48%)	0.96
**Histologic tumour grade**			
Grade 1	38 (45%)	46 (55%)	1.9
Grade 2	108 (54%)	92 (46%)	0.38
Grade 3	103 (53%)	92 (47%)	
**Tubule formation**			
1	20 (51%)	19 (49%)	2.3
2	63 (47%)	72 (53%)	0.3
3	166 (54%)	139 (46%)	
**Polymorphism**			
1	3 (43%)	4 (57%)	2.2
2	75 (47%)	83 (53%)	0.32
3	171 (54%)	143 (46%)	
**Mitosis**			
1	138 (53%)	121 (47%)	1.1
2	48 (54%)	41 (46%)	0.58
3	63 (48%)	68 (52%)	
**Histologic tumour types**			
No special type (NST)	148 (49%)	152 (51%)	
Lobular	33 (65%)	18 (35%)	4.3
Other special types	12 (50%)	12 (50%)	0.22
NST mixed	56 (54%)	48 (46%)	
**Molecular subtypes**			
Luminal A	94 (51%)	89 (49%)	0.7
Luminal B	74 (53%)	67 (47%)	0.87
HER2 enriched.	12 (57%)	9 (43%)	
Triple negative	30 (48%)	33 (52%)	
**Lymph node invasion**			
Absent	158 (51%)	151(49%)	0.25
Present	91 (54%)	79 (46%)	0.62
**Lymhovascular invasion**			
Absent	177 (51%)	176 (49%)	1.8
Present	72 (57%)	54 (43%)	0.1
**Nottingham prognostic index**			
Good prognostic group	86 (50%)	86 (50%)	2.2
Moderate prognostic group	122 (51%)	117 (49%)	0.32

Poor prognostic group	41 (60%)	27 (40%)	
**Ki67 index**			
Low ≤10%	103 (51%)	98 (49%)	0.1
High >10%	75 (49%)	77 (51%)	0.7

Supplementary Table S4. Relationship between combination of nuclear (N)/ cytoplasmic (C) CDK2 co-expression and clinicopathological parameters in invasive breast cancer.

**Categories**	**Negative N - negative C CDK2**	**Positive N - negative C CDK2**	**Positive N - positive C CDK2**	**Positive C – negative N CDK2**	**X^2** ***P*-value**
**Age**					2.2
**<50**	34 (25%)	36 (26%)	38 (28%)	29 (21%)	0.51
**≥50**	104 (30%)	75 (22%)	85 (25%)	78 (23%)	
**Menopausal status**					0.5
**Premenopausal**	45 (29%)	35 (22%)	43 (28%)	33 (21%)	0.91
**Post-menopausal**	93 (28%)	76 (24%)	80 (25%)	74 (23%)	
**Tumour size**					5.7
**≤ 2cm**	92 (32%)	58 (20%)	72 (25%)	67 (23%)	0.12
**> 2cm**	46 (24%)	53 (28%)	51 (27%)	40 (21%)	
**Tumour grade**					
**Grade 1**	25 (30%)	13 (15%)	13 (16%)	33 (39%)	49.8
**Grade 2**	66 (33%)	42 (21%)	38 (19%)	54 (27%)	**<0.0001**
**Grade 3**	47 (24%)	56 (29%)	72 (37%)	20 (10%)	
**Tubule**					
**1**	14 (36%)	6 (15%)	4 (10%)	15 (39%)	18.214
**2**	32 (24%)	31 (23%)	33 (24%)	39 (29%)	**0.006**
**3**	92 (30%)	74 (24%)	86 (28%)	53 (18%)	

**Polymorphism**					
**1**	1 (13%)	2 (29%)	2 (29%)	2 (29%)	33.66
**2**	45 (29%)	30 (19%)	25 (16%)	58 (36%)	**<0.0001**
**3**	92 (29%)	79 (25%)	96 (31%)	47 (15%)	
**Mitosis**					
**1**	87 (33%)	51 (20%)	43 (17%)	78 (30%)	48.6
**2**	22 (25%)	26 (29%)	24 (27%)	17 (19%)	**<0.0001**
**3**	29 (22%)	34 (26%)	56 (43%)	12 (9%)	
**Histologic types**					
**No special type (NST)**	77 (26%)	71 (24%)	96 (32%)	56 (18%)	
**Lobular**	17 (33%)	16 (31%)	6 (12%)	12 (24%)	29.4
**Other special types**	5 (21%)	7 (29%)	2 (8%)	10 (42%)	**0.001**
**Mixed NST**	39 (38%)	17 (16%)	19 (18%)	29 (28%)	
**Molecular subtype**					
**Luminal A**	59 (32%)	35 (19%)	33 (18%)	56 (31%)	27.7
**Luminal B**	38 (27%)	36 (26%)	41 (29%)	26 (18%)	**0.001**
**HER2 +**	6 (29%)	6 (29%)	7 (33%)	2 (9%)	
**Triple negative**	17 (27%)	13 (20%)	27 (43%)	6 (10%)	
**Lymph node status**					
**Negative**	97 (31%)	61 (20%)	77 (25%)	74 (24%)	7.6
**Positive**	41 (24%)	50 (29%)	46 (27%)	33 (20%)	0.05

**Lymphovascular invasion**					
**Absent**	102 (29%)	75 (21%)	85 (24%)	91 (26%)	10.6
**Present**	36 (29%)	36 (29%)	38 (30%)	16 (12%)	**0.01**
**Nottingham Prognostic Index**					
**Good prognostic group**	58 (34%)	28 (16%)	31 (18%)	55 (32%)	30.5
**Moderate prognostic group**	64 (27%)	58 (24%)	72 (30%)	45 (19%)	**<0.0001**
**Poor prognostic group**	16 (24%)	25 (37%)	20 (29%)	7 (10%)	
**Ki67 expression**					9.7
**Low≤14**	64 (32%)	39 (19%)	41 (20%)	57 (29%)	**0.021**
**High>14**	38 (25%)	37 (24%)	48 (32%)	29 (19%)	

HER2, human epidermal growth factor receptor 2 Significant P values are in bold.

Supplementary Table S5. Relationship between combination of nuclear CDK2/P53 co-expression and clinicopathological parameters in invasive breast cancer.

Categories	CDK2-/P53-	CDK2+/P53-	CDK2+ /P53+	CDK2-/P53+	X^2 *P*-value
**Age**					8.8
<50	42 (33%)	35 (28%)	32 (25%)	17 (14%)	**0.03**
≥50	111 (39%)	100 (35%)	40 (14%)	33 (12%)	
**Menopausal status**					2.7
Premenopausal	49 (35%)	42 (30%)	30 (22%)	18 (13%)	0.42
Post-menopausal	104 (39%)	93 (34%)	42 (15%)	32 (12%)	
**Tumour size**					4.6
≤ 2cm	96 (40%)	80 (33%)	35 (14%)	32 (13%)	0.2
> 2cm	57 (34%)	55 (33%)	37 (22%)	18 (11%)	
**Tumour grade**					
Grade 1	41 (59%)	17 (24%)	4 (6%)	8 (11%)	77.1
Grade 2	79 (48%)	58 (35%)	9 (6%)	18 (11%)	**<0.0001**
Grade 3	33 (19%)	60 (34%)	59 (34%)	24 (13%)	
**Tubule**					
1	21 (61%)	5 (15%)	3 (9%)	5 (15%)	28.4
2	50 (44%)	45 (40%)	9 (8%)	9 (8%)	**<0.0001**
3	82 (31%)	85 (32%)	60 (23%)	36 (14%)	
**Polymorphism**					

1	3 (43%)	4 (57%)	0 (0%)	0 (0%)	37.1
2	71 (55%)	37 (28%)	7 (5%)	15 (12%)	**<0.0001**
3	79 (29%)	94 (34%)	65 (24%)	35 (13%)	
**Mitosis**					
1	109 (52%)	66 (31%)	12 (6%)	24 (11%)	65.7
2	24 (31%)	26 (33%)	18 (23%)	10 (13%)	**<0.0001**
3	20 (16%)	43 (36%)	42 (35%)	16 (13%)	
**Histologic types**					
No special type (NST)	78 (30%)	91 (34%)	65 (24%)	32 (12%)	
Lobular	21 (51%)	16 (39%)	2 (5%)	2 (5%)	38.18
Other special types	10 (47%)	6 (29%)	1 (5%)	4 (19%)	**<0.0001**
Mixed NST	44 (54%)	22 (27%)	4 (5%)	12 (14%)	
**Molecular subtype**					
Luminal A	79 (53%)	46 (31%)	10 (7%)	14 (9%)	64.8
Luminal B	40 (32%)	50 (40%)	21 (17%)	15 (11%)	**<0.0001**
Triple negative	11 (20%)	12 (21%)	24 (43%)	9 (16%)	
HER2 +	2 (11%)	4 (22%)	9 (50%)	3 (17%)	
**Lymph node status**					
Negative	108 (41%)	83 (32%)	38 (14%)	35 (13%)	7.9
Positive	45 (31%)	52 (36%)	34 (23%)	15 (10%)	0.04

**Lymphovascular invasion**					
Absent					8.39
Present	123 (42%)	90 (30%)	48 (16%)	37 (12%)	**0.03**
	30 (27%)	45 (40%)	24 (21%)	13 (12%)	
**Nottingham Prognostic Index**					
Good prognostic group					37.87
Moderate prognostic group	74 (52%)	41 (29%)	7 (5%)	20 (14%)	**<0.0001**
Poor prognostic group	67 (31%)	72 (35%)	47 (23%)	22 (11%)	
	12 (20%)	22 (37%)	18 (30%)	8 (13%)	
**Ki67 expression**					
Low≤14	81 (51%)	50 (31%)	15 (9%)	15 (9%)	22.1
High>14	40 (29%)	44 (32%)	37 (27%)	17 (12%)	**<0.0001**

HER2, human epidermal growth factor receptor 2 Significant P values are in bold.

Supplementary Table S6. Clinicopathological significance of nuclear CDK2 and nuclear CDK4 co-expression in breast cancers.

	CDK2-/CDK4-	CDK2+/CDK4+	CDK2+/CDK4-	CDK2-/CDK4+	X2<br>P-value
**Tumour size**					
**≤ 2cm**	50 (63%)	81 (56%)	38 (55%)	90 (65%)	3.46
**> 2cm**	29 (37%)	63 (44%)	31 (45%)	48 (35%)	0.326
**Tumour grade**					
**Grade 1**	9 (11%)	20 (14%)	4 (6%)	41 (30%)	60.21
**Grade2**	43 (55%)	58 (40%)	14 (20%)	63 (45%)	**<0.0001**
**Grade 3**	27 (34%)	66 (46%)	51 (74%)	34 (25%)	
**Tubule formation**					
**1**	6 (8%)	6 (4%)	3 (4%)	19 (14%)	18.86
**2**	21 (26%)	47 (33%)	12 (18%)	45 (33%)	**0.004**
**3**	52 (66%)	91 (63%)	54 (78%)	74 (53%)	
**Pleomorphism**					
**1**	0 (0%)	4 (3%)	0 (0%)	3 (2%)	42.80
**2**	21 (27%)	40 (28%)	8 (12%)	70 (51%)	**<0.0001**
**3**	58 (73%)	100 (69%)	61 (88%)	65 (47%)	
**Mitosis**					
**1**	45 (57%)	71 (49%)	14 (20%)	99 (72%)	52.29
**2**	16 (20%)	27 (19%)	20 (29%)	18 (13%)	**<0.0001**

**3**	18 (23%)	46 (32%)	35 (51%)	21 (15%)	
**Histologic types**					
**No special type (NST)**	55 (70%)	92 (64%)	60 (87%)	68 (49%)	35.25
**Lobular**	8 (10%)	17 (12%)	2 (3%)	14 (10%)	**<0.0001**
**Other special types**	5 (6%)	7 (5%)	1 (1%)	9 (7%)	
**Mixed NST**	11 (14%)	28 (19%)	6 (9%)	47 (34%)	
**Lymph node status**					
**Absent**	51 (65%)	82 (57%)	43 (62%)	99 (72%)	6.79
**Present**	28 (35%)	62 (43%)	26 (38%)	39 (28%)	0.079
**Lymphovascular invasion**					
**Absent**	62 (79%)	97 (67%)	46 (67%)	109 (79%)	7.44
**Present**	17 (21%)	47 (33%)	23 (33%)	29 (21%)	0.059
**Nottingham Prognostic index**					
**Good prognostic group**	29 (37%)	41 (28%)	12 (17%)	69 (50%)	28.73
**Moderate prognostic group**	43 (54%)	77 (54%)	44 (64%)	56 (41%)	**<0.0001**
**Poor prognostic group**	7 (9%)	26 (18%)	13 (19%)	13 (9%)	
**ER status**					
**Negative**	15 (19%)	24 (17%)	24 (35%)	16 (12%)	16.68
**Positive**	64 (81%)	119 (83%)	45 (65%)	121 (88%)	**0.001**
**PgR status**					
**Negative**	25 (32%)	48 (34%)	35 (51%)	47 (34%)	8.01

**Positive**	52 (68%)	95 (66%)	33 (49%)	91 (66%)	0.046
**HER2 status**					
**Negative**	70 (89%)	127 (89%)	53 (77%)	127 (92%)	10.32
**Positive**	9 (11%)	16 (11%)	16 (23%)	11 (8%)	0.016
**Ki67 expression**					
**Low≤14**	30 (54%)	58 (53%)	14 (33%)	80 (71%)	20.02
**High>14**	26 (46%)	51 (47%)	28 (67%)	32 (29%)	**<0.0001**
**Molecular subtypes**					
**Luminal A**	28 (42%)	54 (44%)	76 (63%)	9 (15%)	41.67
**Luminal B**	27 (41%)	45 (37%)	30 (25%)	27 (46%)	**<0.0001**
**Triple negative**	9 (14%)	18 (14%)	10 (8%)	17 (29%)	
**HER2 +**	2 (3%)	6 (5%)	5 (4%)	6 (10%)	
**Menopausal status**					
**Premenopausal**	30 (38%)	48 (33%)	27 (39%)	36 (26%)	5.05
**Postmenopausal**	49 (62%)	96 (67%)	42 (61%)	102 (74%)	0.168
**Age at diagnosis**					
**<50**	29 (37%)	48 (33%)	23 (33%)	26 (19%)	11.05
**≥50**	50 (63%)	96 (67%)	46 (67%)	112 (81%)	0.011

Supplementary Table S7. Clinicopathological significance of nuclear CDK2 and nuclear CDK6 co-expression in breast cancers.

	CDK2-/CDK6-	CDK2+/CDK6+	CDK2+/CDK6-	CDK2-/CDK6+	X^2 *P*-value
**Tumour size**					
**≤ 2cm**	90 (63%)	39 (65%)	71 (53%)	42 (64%)	4.75
**> 2cm**	52 (37%)	21 (35%)	64 (47%)	24 (36%)	0.191
**Tumour grade**					
**Grade 1**	23 (16%)	15 (25%)	9 (7%)	23 (35%)	68.75
**Grade2**	73 (52%)	25 (42%)	34 (25%)	30 (45%)	**<0.0001**
**Grade 3**	46 (32%)	20 (33%)	92 (68%)	13 (20%)	
**Tubule formation**					
**1**	11 (8%)	5 (8%)	5 (4%)	11 (17%)	15.82
**2**	46 (32%)	22 (37%)	32 (24%)	18 (27%)	**0.015**
**3**	85 (60%)	33 (55%)	98 (72%)	37 (56%)	
**Pleomorphism**					
**1**	1 (1%)	3 (5%)	1 (1%)	2 (3%)	52.69
**2**	47 (33%)	25 (42%)	18 (13%)	38 (58%)	**<0.0001**
**3**	94 (66%)	32 (53%)	116 (86%)	26 (39%)	
**Mitosis**					
**1**	86 (61%)	36 (60%)	38 (28%)	50 (76%)	56.46
**2**	27 (19%)	11 (18%)	32 (24%)	8 (12%)	**<0.0001**
**3**	29 (20%)	13 (22%)	65 (48%)	8 (12%)	
**Histologic types**					
**No special type (NST)**	95 (67%)	27 (45%)	109 (81%)	20 (30%)	59.07
**Lobular**	11 (8%)	9 (15%)	8 (6%)	11 (17%)	**<0.0001**
**Other special types**	7 (5%)	6 (10%)	2 (1%)	6 (9%)	
**Mixed NST**	29 (20%)	18 (30%)	16 (12%)	29 (44%)	
**Lymph node status**					
**Absent**	92 (65%)	34 (57%)	78 (58%)	49 (74%)	6.36
**Present**	50 (35%)	26 (43%)	57 (42%)	17 (26%)	0.095
**Lymphovascular invasion**					

**Absent**	106 (75%)	37 (62%)	93 (69%)	57 (86%)	11.14
**Present**	36 (25%)	23 (38%)	42 (31%)	9 (14%)	**0.011**
**Nottingham Prognostic index**					
**Good prognostic group**	56 (40%)	25 (42%)	21 (16%)	36 (54%)	37.60
**Moderate prognostic group**	70 (49%)	26 (43%)	87 (64%)	25 (38%)	**<0.0001**
**Poor prognostic group**	16 (11%)	9 (15%)	27 (20%)	5 (8%)	
**ER status**					
**Negative**	23 (16%)	9 (15%)	39 (29%)	5 (8%)	15.57
**Positive**	118 (84%)	51 (85%)	95 (71%)	60 (92%)	**0.001**
**PgR status**					
**Negative**	50 (36%)	23 (38%)	57 (43%)	20 (30%)	3.29
**Positive**	90 (64%)	37 (62%)	76 (57%)	46 (70%)	0.349
**HER2 status**					
**Negative**	125 (88%)	53 (88%)	113 (84%)	61 (92%)	2.78
**Positive**	17 (12%)	7 (12%)	21 (16%)	5 (8%)	0.427
**Ki67 expression**					
**Low≤14**	62 (57%)	33 (70%)	37 (39%)	42 (84%)	30.20
**High>14**	46 (43%)	14 (30%)	57 (61%)	8 (16%)	**<0.0001**
**Molecular subtypes**					
**Luminal A**	59 (48%)	30 (57%)	29 (25%)	41 (74%)	46.19
**Luminal B**	46 (37%)	14 (26%)	49 (42%)	9 (16%)	**<0.0001**
**Triple negative**	12 (10%)	7 (13%)	28 (24%)	3 (6%)	
**HER2 +**	6 (5%)	2 (4%)	10 (9%)	2 (4%)	
**Menopausal status**					
**Premenopausal**	46 (32%)	21 (35%)	49 (36%)	17 (26%)	2.36
**Postmenopausal**	96 (68%)	39 (65%)	86 (64%)	49 (74%)	0.501
**Age at diagnosis**					
**<50**	37 (26%)	16 (27%)	51 (38%)	13 (20%)	8.57
**≥50**	105 (74%)	44 (73%)	84 (62%)	53 (80%)	**0.035**

Supplementary Table S8. Relationship between nuclear CDK2 and clinicopathological parameters in the DCIS

Categories	Negative /low H. score ≤5	Positive /high H. score ≤5	X² *P*-value
**Age**			1.4 0.233
<50	26 (44%)	33 (56%)	
≥50	84 (53%)	74 (47%)	
**Tumour size**			3.1 0.07
≤2cm	51 (58%)	37 (42%)	
>2cm	59 (46%)	70 (54%)	
**Tumour grade**			0.48 0.78
Low grade	16 (55%)	13 (45%)	
Intermediate grade	30 (48%)	33 (52%)	
High grade	64 (51%)	61 (49%)	
**Molecular subtype**			5.1 0.16
Luminal A	55 (61%)	35 (39%)	
Luminal B	15 (50%)	15 (50%)	
HER2+	11 (41%)	16 (59%)	
Triple Negative	6 (40%)	9 (60%)	
**Comedo necrosis**			1.3 0.25
Negative	41 (56%)	32 (44%)	
Positive	69 (48%)	75 (52%)	
ER Status			3.2 0.07
Negative	19 (42%)	26 (58%)	
Positive	80 (58%)	59 (42%)	
**PR Status**			6.9

			**0.009**
Negative	30 (42%)	42 (58%)	
Positive	70 (61%)	44 (39%)	
**HER2 Status**			0.91
			0.34
Negative	86 (56%)	68 (44%)	
Positive	21 (48%)	23 (52%)	
**Recurrence**			1.1
			0.3
No recurrence	96 (49%)	98 (51%)	
Recurrence	14 (61%)	9 (39%)	
**Ki67 expression**			14.238
			**<0.0001**
Low (≤14)	85 (63%)	49 (37%)	
High (>14)	14 (31%)	31 (69%)	

HER2, human epidermal growth factor receptor 2 Significant P values are in bold

Supplementary Table S9. Relationship between cytoplasmic CDK2 and clinicopathological parameters in the DCIS

Categories	Negative /low H. score ≤20	Positive /high H. score ≤20	X² *P*-value
**Age**			0.4<br>0.52
<50	32 (54%)	27 (46%)	
≥50	77 (49%)	79 (51%)	
**Tumour size**			0.09<br>0.75
≤2cm	43 (49%)	44 (51%)	
>2cm	66 (52%)	62 (48%)	
**Tumour grade**			16.4<br>**<0.0001**
Low grade	7 (24%)	22 (76%)	
Intermediate grade	25 (41%)	36 (59%)	
High grade	77 (62%)	48 (38%)	
**Molecular subtype**			9.9<br>**0.01**
Luminal A	35 (40%)	53 (60%)	
Luminal B	18 (60%)	12 (40%)	
HER2+	19 (70%)	8 (30%)	
Triple Negative	9 (60%)	6 (40%)	
**Comedo necrosis**			9.2<br>**0.002**
Negative	26 (36%)	46 (64%)	

Positive	83 (58%)	60 (42%)	
**ER Status**			6.6 **0.01**
Negative	30 (67%)	15 (33%)	
Positive	61 (44%)	76 (56%)	
**PR Status**			9.9 **0.002**
Negative	46 (64%)	26 (36%)	
Positive	45 (40%)	67 (60%)	
**HER2 Status**			3.3 0.07
Negative	105 (65%)	56 (35%)	
Positive	35 (79%)	9 (21%)	
**Recurrence**			1.1 0.29
No recurrence	136 (68%)	65 (32%)	
Recurrence	18 (78%)	5 (22%)	
**Ki67 expression**			3.4 0.06
Low (≤14)	61 (46%)	71 (54%)	
High (>14)	28 (62%)	17 (38%)	

Supplementary Table S10. Relationship between nuclear(N)/ cytoplasmic (C) CDK2 co-expression and clinicopathological parameters in the DCIS cohort

Categories	Negative N - negative C CDK2	Positive N - negative C CDK2	Positive N - positive C CDK2	Positive C - negative N CDK2	X^2 *P*-value
**Age**					
<50	20 (34%)	15 (25%)	11 (19%)	13 (22%)	1.4
≥50	47 (30%)	35 (23%)	27 (17%)	47 (30%)	0.71
**Tumour size**					3.56 **0.3**
≤2cm	30 (34%)	16 (18%)	13 (15%)	28 (33%)	
>2cm	37 (28%)	34 (27%)	25 (20%)	32 (25%)	
**Tumour grade**					12.9 **0.043**
Low grade	6 (20%)	4 (14%)	8 (28%)	11 (38%)	
Intermediate grade	16 (26%)	11 (18%)	15 (25%)	19 (31%)	
High grade	45 (36%)	35 (28%)	15 (12%)	30 (24%)	
**Molecular subtype**					19.87 **0.01**
Luminal A	27 (31%)	12 (14%)	14 (15%)	35 (40%)	
Luminal B	11 (37%)	7 (23%)	7 (23%)	5 (17%)	
HER2+	7 (26%)	12 (44%)	3 (11%)	5 (19%)	
Triple Negative	5 (34%)	6 (40%)	2 (13%)	2 (13%)	
**Comedo type necrosis**					9.4 **0.024**
Negative	21 (29%)	9 (13%)	16 (22%)	26 (36%)	
Positive	46 (32%)	41 (29%)	22 (15%)	34 (24%)	
**ER Status**					13.5

					**0.004**
Negative	14 (31%)	18 (40%)	5 (11%)	8 (18%)	
Positive	45 (33%)	21 (15%)	26 (19%)	45 (33%)	
**PR Status**					14.2<br>**0.003**
Negative	25 (34%)	23 (32%)	12 (17%)	12 (17%)	
Positive	33 (29%)	16 (14%)	19 (17%)	44 (40%)	
**HER2 Status**					4.930<br>0.177
Negative	91 (35%)	129 (50%)	31 (12%)	9 (3%)	
Positive	41 (48%)	35 (41%)	7 (8%)	2 (3%)	
**Recurrence**					1.07<br>0.62
No recurrence	58 (30%)	47 (24%)	34 (18%)	53 (28%)	
Recurrence	9 (39%)	3 (13%)	4 (18%)	7 (30%)	
**Ki67 expression**					18.3<br>**<0.0001**
Low	45 (34%)	20 (15%)	18 (14%)	49 (37%)	
High	11 (24%)	18 (40%)	10 (22%)	6 (14%)	

HER2, human epidermal growth factor receptor 2 Significant P values are in bold.

Supplementary Figure 1

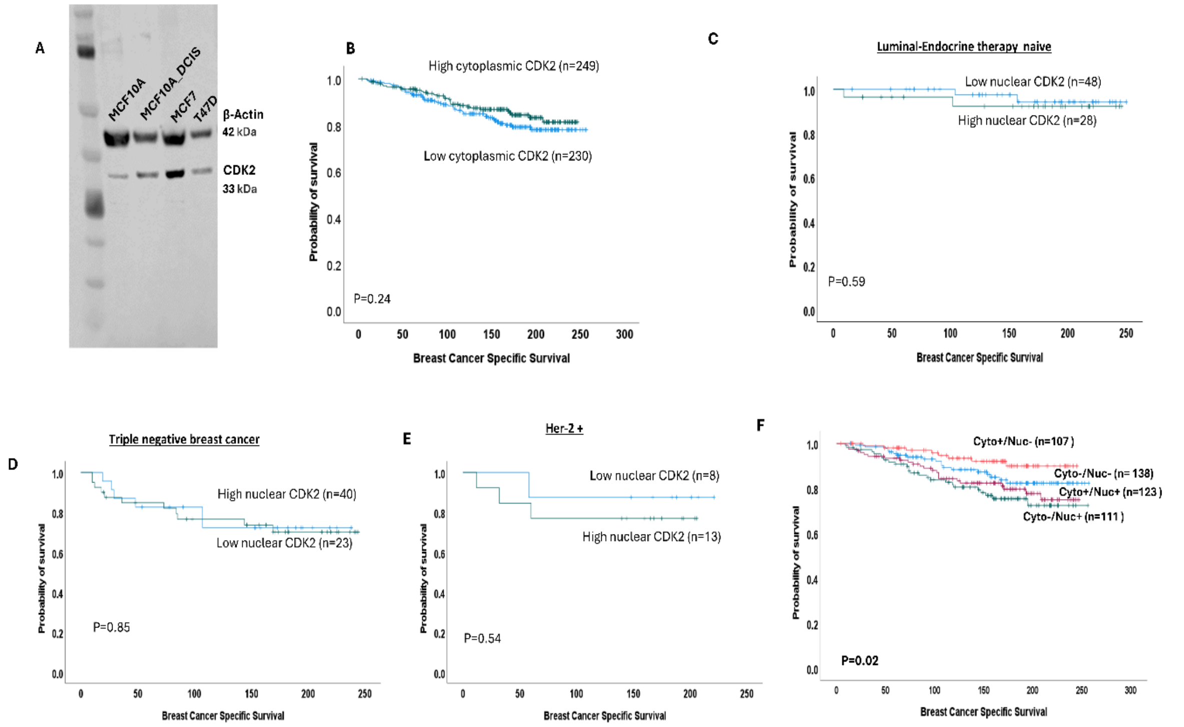

Supplementary Figure S1: CDK2 expression in BC. (A) Western blot of CDK2 antibody shows specific bands at 33 KDa. **(B)** Kaplan-Meier curve for CDK2 cytoplasmic expression and BCSS in the whole cohort. **(C)** Kaplan-Meier curve for CDK2 nuclear expression and BCSS in the endocrine therapy naive patients. **(D)** Kaplan-Meier curve for CDK2 nuclear expression and BCSS in triple negative BC. **(E)** Kaplan-Meier curve for CDK2 nuclear expression and BCSS in HER2 enriched BC. **(F)** Kaplan-Meier curve for CDK2 nuclear/ cytoplasmic co-expression and BCSS in the whole cohort.

Supplementary Figure 2

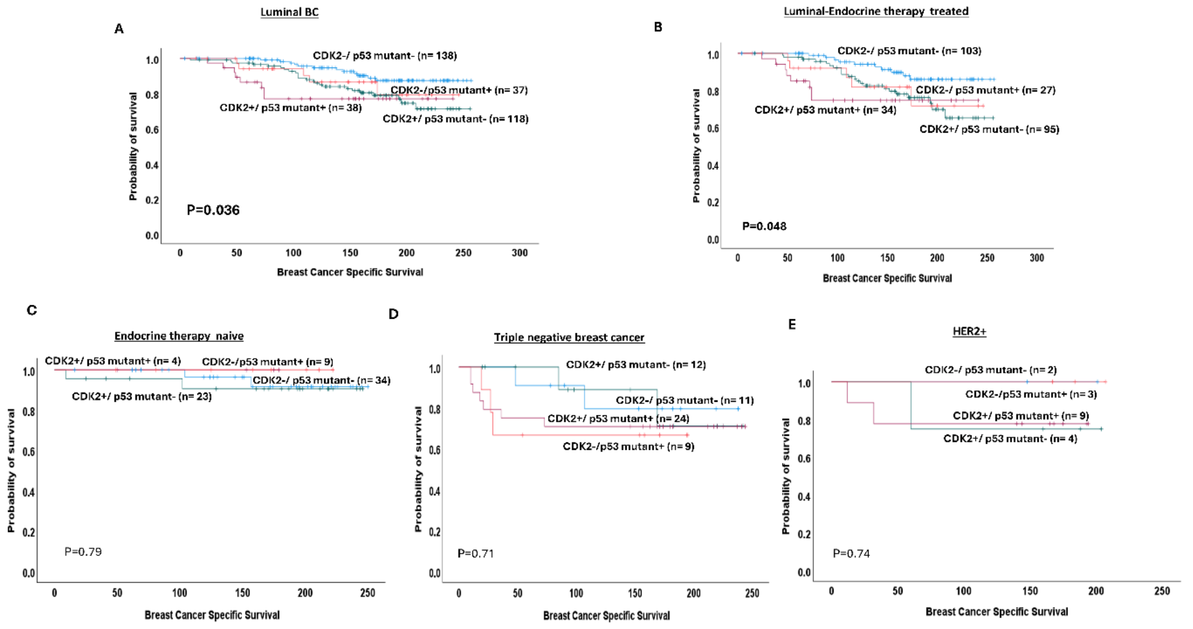

Supplementary figure S2: CDK2/p53 co-expression in BC (A) Kaplan-Meier curve for CDK2/p53 co-expression and BCSS in luminal BC. **(B)** Kaplan-Meier curve for CDK2/p53 co-expression and BCSS in the endocrine therapy treated patients. **(C)** Kaplan-Meier curve for CDK2/p53 co-expression and BCSS in the endocrine therapy naive patients. **(D)** Kaplan-Meier curve for CDK2/p53 co-expression and BCSS in triple negative BC. **(E)** Kaplan-Meier curve for CDK2/p53 co-expression and BCSS in HER2 enriched BC.

Supplementary Figure 3

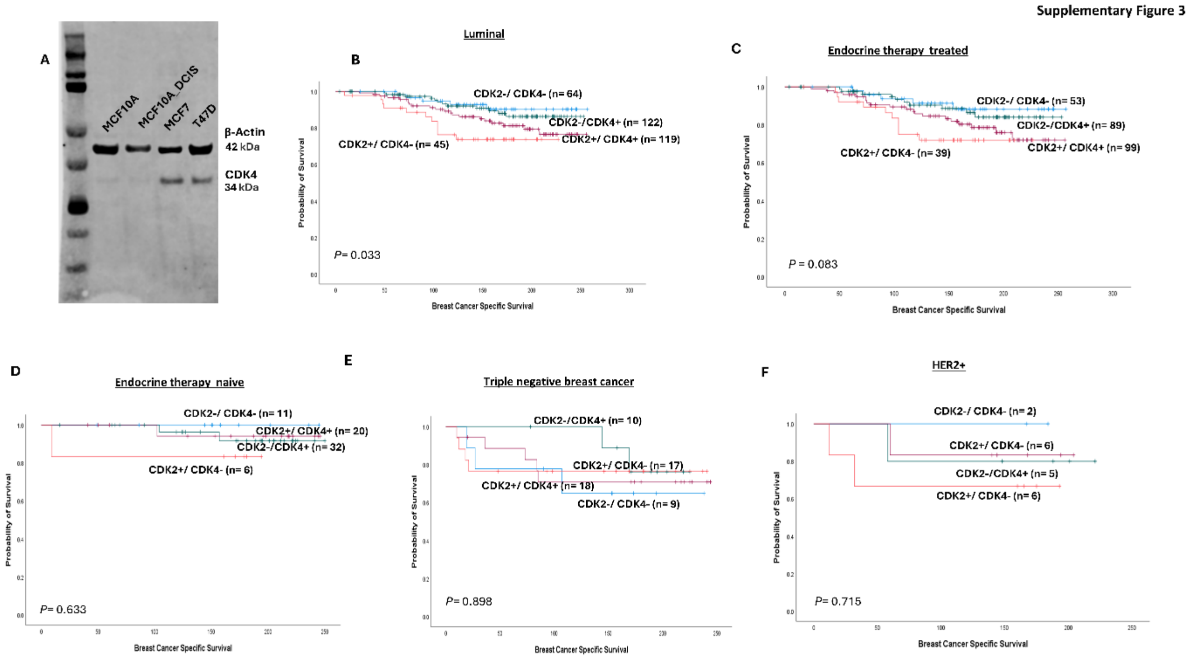

Supplementary figure S3: CDK2/CDK4 co-expression in BC. (A) Western blot of CDK4 antibody shows specific bands at 34 KDa. **(B)** Kaplan-Meier curve for CDK2/CDK4 co-expression and BCSS in the luminal BC. **(C)** Kaplan-Meier curve for CDK2/CDK4 co-expression and BCSS in the endocrine therapy treated patients. **(D)** Kaplan-Meier curve for CDK2/CDK4 co-expression and BCSS in the endocrine therapy naïve patients. **(E)** Kaplan-Meier curve for CDK2/CDK4 co-expression and BCSS in triple negative BC. **(F)** Kaplan-Meier curve for CDK2/CDK4 co-expression and BCSS in the HER2 enriched BC.

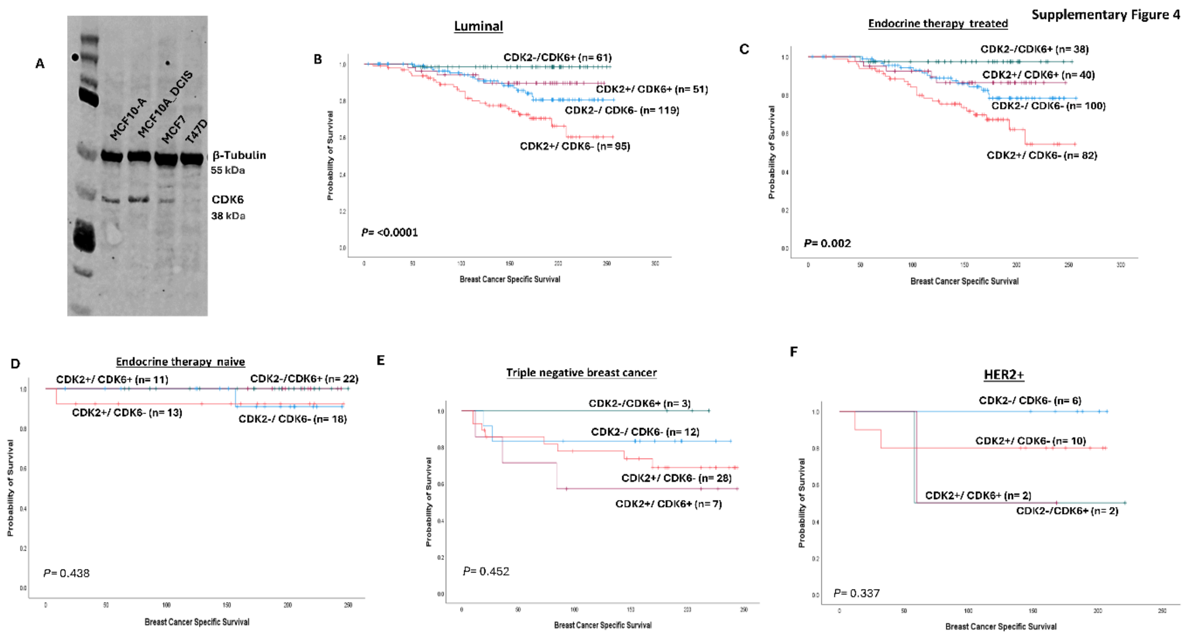

Supplementary figure S4: CDK2/CDK4 co-expression in BC. (A) Western blot of CDK6 antibody shows specific bands at 38 KDa. **(B)** Kaplan-Meier curve for CDK2/CDK6 co-expression and BCSS in luminal BC. **(C)** Kaplan-Meier curve for CDK2/CDK6 co-expression and BCSS in the endocrine therapy treated patients. **(D)** Kaplan-Meier curve for CDK2/CDK6 co-expression and BCSS in the endocrine therapy naïve patients. **(E)** Kaplan-Meier curve for CDK2/ CDK6 co-expression and BCSS in triple negative BC. **(F)** Kaplan-Meier curve for CDK2/CDK6 co-expression and BCSS in HER2 enriched BC.

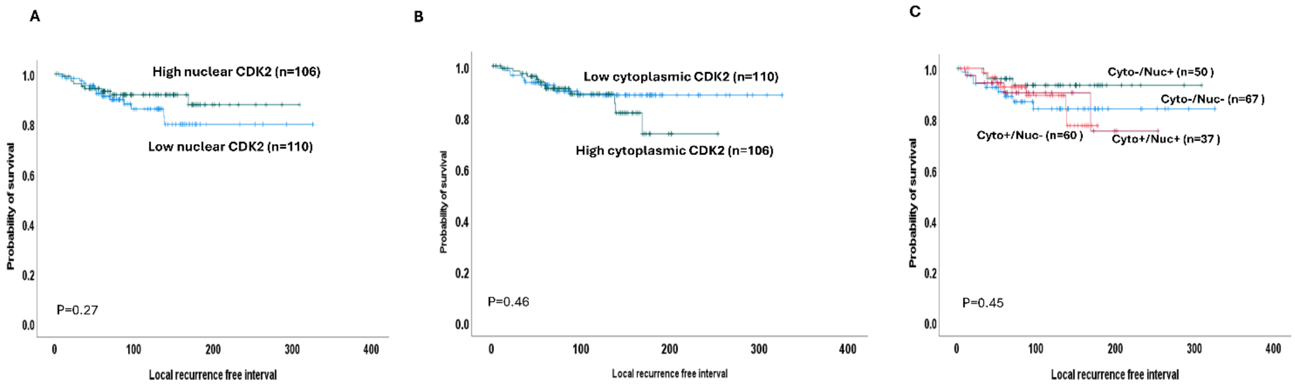

Supplementary figure S5: CDK2 expression in DCIS. (A) Kaplan-Meier curve for nuclear CDK2 expression in DCIS. **(B)** Kaplan-Meier curve for cytoplasmic CDK2 expression in DCIS. **(C)** Kaplan-Meier curve for CDK2 nuclear/cytoplasmic co-expression in DCIS.
